# Construction and Evaluation of a Ketosis Prediction Model Based on Milk MIR Spectra and Feature Wavenumber Selection in Dairy Cows

**DOI:** 10.3390/ani16203196

**Published:** 2026-10-12

**Authors:** Xiaoli Ren, Zhuo Yang, Aote Han, Yikai Fan, Yi Zhang, Shaohu Chen, Zhen Zhang, Lei Yan, Zheng Cao, Wenjiao Liu, Xiaofeng Huang, Dongwei Wang, Guixiang Zhang, Qingxia Yan, Shujun Zhang

**Affiliations:** 1 Frontiers Science Center for Animal Breeding and Sustainable Production, Huazhong Agricultural University, Ministry of Education, Wuhan 430070, China; renxiaoli@webmail.hzau.edu.cn (X.R.); yang_zhuo@webmail.hzau.edu.cn (Z.Y.); hat@webmail.hzau.edu.cn (A.H.); fanyikai123@webmail.hzau.edu.cn (Y.F.); huang_xiaofeng@webmail.hzau.edu.cn (X.H.); wangdwei@webmail.hzau.edu.cn (D.W.); 2 Henan Provincial International Joint Laboratory for Dairy Health Farming, Henan Dairy Herd Improvement Center, Zhengzhou 450046, China; 3 Information Technology Research Center, Beijing Academy of Agriculture and Forestry Sciences, Beijing 100097, China; 4 College of Animal Science and Technology, China Agricultural University, Beijing 100193, China; yizhang@cau.edu.cn; 5 Dairy Association of China, Beijing 100193, China; net@dac.org.cn (S.C.); caozheng@163.com (Z.C.); lwjsmile@163.com (W.L.); 6 Henan Seed Industry Development Center, Zhengzhou 450046, China; zzgxu@163.com (Z.Z.); yanleihcy@163.com (L.Y.); 7 National Animal Husbandry Service, Beijing 100125, China; 1350999617@ag.com

**Keywords:** dairy cows, ketosis, mid-infrared spectra, phenotypic detection models, machine learning

## Abstract

Ketosis is a common metabolic disorder in dairy cows and occurs mainly during early lactation. Blood β-hydroxybutyrate (BHB) is widely used for diagnosis, but large-scale testing is limited by sampling requirements and cost. We therefore studied lactating Holstein cows from 12 large-scale dairy farms in central China. Milk samples, milk composition traits, and milk mid-infrared (MIR) spectra were collected through the Dairy Herd Improvement (DHI) testing system from April 2019 to December 2022, and ketosis diagnoses were obtained from farm health records; samples were labeled as healthy or ketotic based on the temporal relationship between diagnosis and sampling. A total of 2206 samples were used to build the calibration and validation datasets, in which five feature wavenumber selection methods and five machine learning algorithms were compared. Ketotic cows had a higher milk fat percentage and fat-to-protein ratio and a lower protein and lactose percentage (*p* < 0.01). The best combination was PLS-DA based on 104 CARS-selected wavenumbers, which achieved a sensitivity of 0.83, a specificity of 0.79, a balanced accuracy of 0.81 and an area under the receiver operating characteristic curve (AUC) of 0.88 in validation. MIR spectroscopy combined with machine learning can therefore support large-scale ketosis phenotyping within the DHI system.

## Simple Summary

Ketosis is a common metabolic disease in dairy cows that reduces milk production and impairs reproductive performance, causing significant economic losses. Early detection is challenging because current diagnostic methods require blood sampling, which is costly and difficult to implement on a large scale. In this study, we developed a non-invasive screening method that combines milk mid-infrared spectra—a technology already used in routine milk testing—with machine learning algorithms. We analyzed 2206 milk samples from 12 farms in China and built a predictive model that achieved 83% sensitivity and 79% specificity using only 104 spectral wavenumbers. This approach enables large-scale, low-cost ketosis monitoring within existing Dairy Herd Improvement testing programs, supporting early disease detection, improved herd health, reduced economic losses and genetic selection for disease resistance in dairy cows.

## 1. Introduction

Ketosis is a common metabolic disorder in dairy cows, arising from negative energy balance and typically manifesting during the perinatal period and early lactation, with an incidence of 10–30% [1]. The disease impairs milk production and reproductive efficiency and increases the risk of abomasal displacement, metritis, lameness and culling [1,2], leading to decreased yield [3] and reduced conception rates [4]. Economic losses have been estimated at $77–203 per case [5], underscoring the need for cost-effective, high-throughput screening tools for early detection and genetic improvement for disease resilience.

Ketosis includes clinical and subclinical forms, both of which are characterized by elevated ketone bodies in blood, milk or urine [6]. Although blood BHB is the diagnostic gold standard, its large-scale application is limited by sampling, cost and logistics, prompting interest in DHI-compatible alternatives [7]. Milk MIR spectroscopy provides rapid, automated, non-destructive analysis of milk composition and spectral changes can serve as biomarkers for metabolic disorders [8,9]. Beyond metabolic traits, MIR-based diagnostic models have also been developed for udder health, underscoring the broader phenotyping potential of milk spectra [10]. Previous MIR models for predicting acetone, acetoacetate or BHB, or for detecting ketosis using the fat-to-protein ratio and other traits, have achieved moderate performance, with sensitivity ranging from 0.69 to 0.75 and specificity from 0.73 to 0.95 [11,12,13]. However, these models rely on pre-selected variables rather than full-spectrum data and unassigned wavenumber regions (1701–2797 and 3734–5011 cm^−1^) may carry additional metabolic information relevant to ketosis [14,15,16].

Therefore, the objective of this study was to develop and evaluate a milk MIR-based ketosis prediction approach for Holstein cows and to determine whether feature wavenumber selection could improve discrimination between healthy and ketotic animals. We further examined the biological relevance of the selected wavenumbers and the potential of the resulting model for high-throughput phenotyping within routine DHI testing.

## 2. Materials and Methods

**Figure 1:**
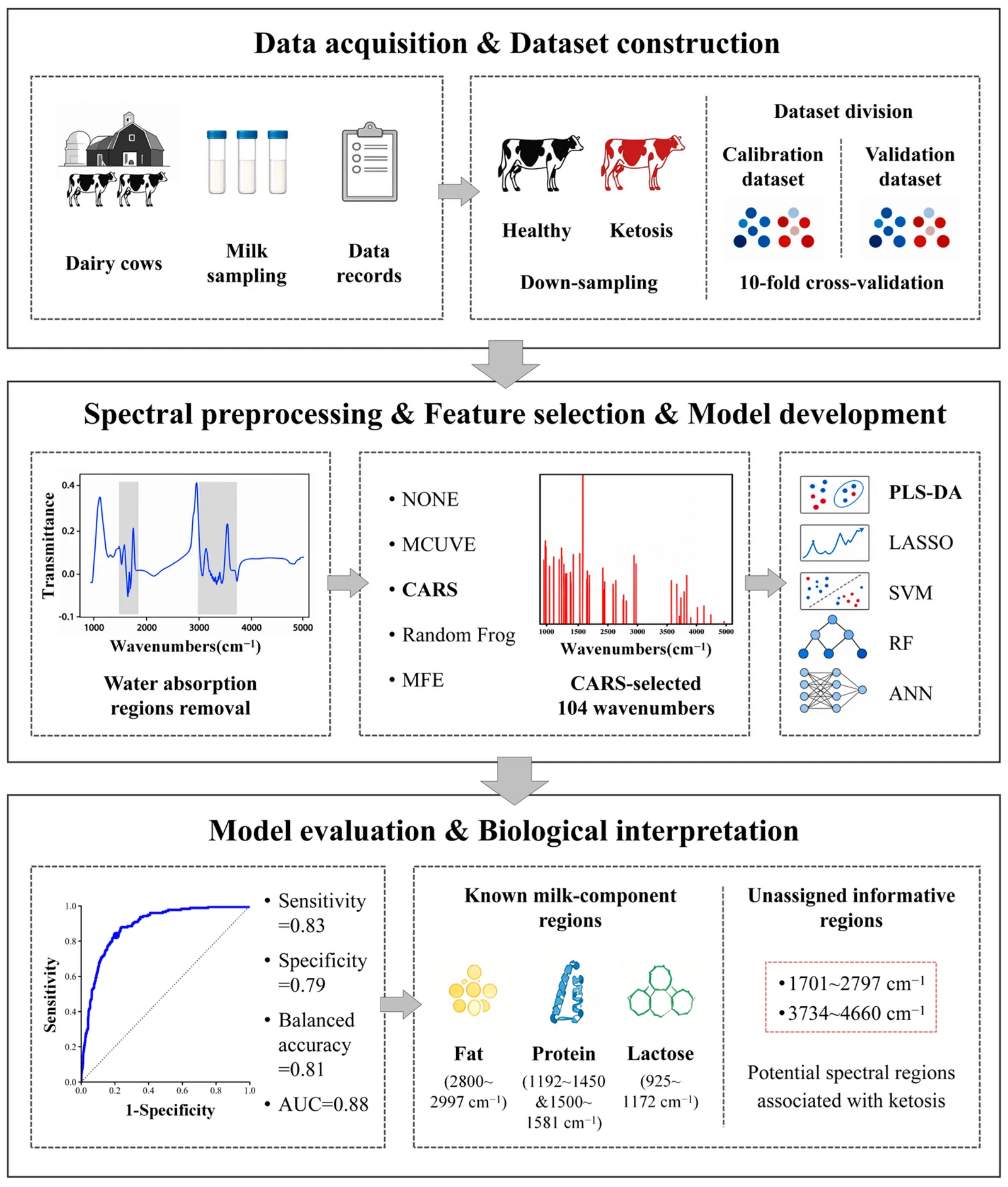
Overall experimental workflow of the ketosis prediction model in dairy cows. In this study, we collected health diagnosis records of dairy cows. Additionally, milk composition data, somatic cell count data and MIR spectral data were obtained from milk samples, which were then classified into healthy and ketotic samples. Five spectral feature selection methods—namely NONE (no feature selection), MCUVE, CARS, Random Frog and MFE—and five machine learning algorithms—PLS-DA, LASSO, SVM, RF and ANN—were applied to construct the models. Model performance was evaluated using sensitivity, specificity and balanced accuracy. The ROC curve of the final model and its AUC are also shown in this figure. Arrows indicate the sequential flow of data processing and analysis steps. Colors are used only for visual distinction and do not encode additional information. The flowchart layout and overall design were manually created by the authors. The decorative icons in this figure were generated using ChatGPT (OpenAI, ChatGPT Images 2.0) as visual aids. The authors take full responsibility for the content of the final figure.

The overall experimental design and analysis process of this study are shown in Figure 1. No copyrighted third-party materials were used in the preparation of this figure.

### 2.1. Sample Collection and Analysis

This study focused on lactating Holstein dairy cows raised on 12 dairy farms in central China. The dairy farms are located in a warm-temperate to subtropical, humid to semi-humid monsoon climate environment. The feed was a total mixed ration (TMR) and drinking water was supplied by automatic waterers installed in the cow sheds. Management was highly standardized, and records of health and production information were complete.

#### 2.1.1. Collection of Ketosis-Related Disease Data

We collected health diagnosis records from the 12 dairy farms. Ketosis-related diagnosis data included farm name, cow number, parity at illness, disease category, disease name, date of illness, diagnosing veterinarian, treatment date, recovery date and recovery outcome. In most cases, ketosis was detected by measuring the blood β-hydroxybutyrate (BHB) concentration of postpartum cows within 100 days in milk with a handheld meter, with the measurement being performed by the farm veterinarians. The health records contain the diagnostic outcome (ketotic or not ketotic), but not the numerical BHB value, the threshold applied or the meter brand; the diagnosis therefore followed each farm’s routine protocol, and the records do not allow us to establish whether all 12 farms used the same threshold or the same type of meter.

#### 2.1.2. Collection and Measurement of Dairy Cow Samples

From April 2019 to December 2022, we regularly collected milk samples from lactating cows according to the guidelines of the International Committee for Animal Recording (ICAR). The volume of each collected milk sample was between 30 mL and 50 mL. After collection, we immediately mixed samples with a preservative (2-bromo-2-nitro-1,3-propanediol) and transported them to the DHI laboratory (Zhengzhou, China). We analyzed the samples using a FOSS milk composition analyzer (FOSS, Hillerød, Denmark), which employed Fourier transform mid-infrared spectrometry (FT-MIR). After analysis, we obtained data on milk components (including milk protein percentage, milk fat percentage and lactose percentage), somatic cell count and differential somatic cell count, as well as MIR data consisting of 1060 wavenumber points ranging from 925 to 5011 cm^−1^. The dataset comprised 18,331 milk samples with complete records from 4828 dairy cows across 12 farms, including 14,632 samples from 2966 healthy cows and 3699 samples from 1862 cows with a history of ketosis.

### 2.2. Data Processing and Analysis

- Grouping of dairy cow health status and division of MIR calibration dataset

We extracted ketosis records for each cow per lactation. For cows with one or more illness records, we identified the illness and recovery dates for each episode and calculated the first illness date and last recovery date within each lactation. We retained only illness records with onset after calving (i.e., records where both the first illness date and the last recovery date occurred after the calving date). We determined the health status of each cow at the time of milk sampling based on the first illness date, last recovery date and sample collection date. Specifically: (a) if the sample collection date was after the first illness date but before the last recovery date, we classified the cow as “Ketosis” at sampling; (b) if the sample collection date was 150 days (inclusive) before the first illness date, we classified the cow as “Pre-illness”; (c) if the sample collection date was 150 days (inclusive) after the last recovery date, we classified the cow as “Recovered”; and (d) we classified cows that never suffered from ketosis or any other disease as “Healthy”. Based on the above classification, there were 14,632 samples from healthy cows, 644 from ketotic cows, 191 from pre-illness cows and 2864 from recovered cows. Cows ranged from the first to sixth parity, and days in milk ranged from 1 to 500 d.

- Division of MIR calibration dataset

For model development, 191 pre-illness and 2864 recovered samples were excluded, leaving 15,276 samples (14,632 healthy and 644 ketotic). Then because ketosis mainly occurs in early lactation, we retained only samples from cows with days in milk ≤100 d; this excluded 10,714 healthy and 45 ketotic samples and left 3918 healthy and 599 ketotic samples. The healthy and ketotic samples were then each divided into calibration and validation datasets at a ratio of 7:3, yielding 2736 healthy and 425 ketotic samples in the calibration dataset and 1182 healthy and 174 ketotic samples in the validation dataset. To further balance the number of healthy and ketotic samples, we applied random downsampling to the calibration dataset so that the numbers of healthy and ketotic samples were equal. The final dataset comprised 2206 samples, consisting of 850 samples in the calibration dataset (425 healthy and 425 ketotic) and 1356 samples in the validation dataset (1182 healthy and 174 ketotic). These samples were obtained from 1571 cows, of which 1081 were classified as healthy cows and 490 as ketotic cows. In total, 16,125 samples (13,025 healthy, 45 ketotic, 191 pre-illness and 2864 recovered) were excluded. Samples with missing values for MIR spectra, milk composition traits or somatic cell count had been removed during routine DHI data curation, before the 18,331 samples were assembled; all of the 18,331 samples therefore had complete records for these variables, and no further observations were excluded because of missing values.

- Mid-infrared spectroscopy data processing

MIR absorbance values were converted to transmittance according to the Beer–Lambert law [17].

- Analysis of the influence of health status on daily milk yield, milk composition and somatic cell count

We used the lme4 package [18] in R software (version 4.3.0) [19] to analyze the effects of health status on daily milk yield, milk composition and somatic cell count in the model development and validation datasets. Multiple comparisons between healthy and ketotic cows were conducted using the lsmeans package [20] in R software [19].

*y_ijklmn_* = *H_i_* + *Par_j_* + *TM_k_* + *Health_l_* + *a_m_* + *e_ijklmn_* (1)

where *y_ijklmn_* represents the dependent variable (e.g., daily milk yield, milk composition, or somatic cell count); *H_i_* is the herd effect; *Par_j_* is the parity effect; *TM_k_* is the effect of sample measurement month; *Health_l_* is health status (0 = healthy, 1 = ketotic); *a_m_* is the random effect of the individual cow; and *e_ijklmn_* is the residual.

### 2.3. Model Development and Evaluation

No additional spectral preprocessing was applied. The aim of this study was to evaluate whether ketosis can be detected from milk MIR spectra as they are routinely produced by the DHI testing system; our focus was therefore on comparing the effects of different feature wavenumber selection methods and modeling algorithms.

- Feature extraction from MIR spectra

We used five methods for feature extraction from MIR spectra. To avoid interference from the water absorption regions, we first removed these regions and retained 875 MIR wavenumbers in the ranges of 925.92–1581.78 cm^−1^, 1701.38–3028.53 cm^−1^ and 3630.38–5011.54 cm^−1^ (denoted as NONE). On this basis, we further compared feature selection algorithms such as MCUVE [21], CARS [22], Random Frog [23] and MFE [24]. CARS was run ten times independently on the calibration dataset; within each run, the wavenumbers are eliminated over successive iterations, and the subset with the lowest cross-validated RMSECV obtained in that run was taken as its selection. The ten runs are provided as Supplementary Data S1 (875 candidate wavenumbers × 10 runs), and the first run, whose selection retained 104 wavenumbers, is the one used for the final model and listed in Supplementary Table S1.

- Model development

We employed five machine learning algorithms for model development, including PLS-DA, LASSO, SVM, RF and ANN.

- Model evaluation

We subjected the calibration dataset to 10-fold cross-validation, in which the hyperparameters of each algorithm were optimized over the grids given in Supplementary Table S2. Specifically, we divided the calibration dataset into a training set and a test set at a ratio of 9:1 based on the sample size for model development and internal validation. The evaluation metrics for internal and external validation of the models included sensitivity, specificity and balanced accuracy, defined as the mean of sensitivity and specificity. For the final model, the positive predictive value, negative predictive value, F1-score, Matthews correlation coefficient, AUC and precision–recall AUC (PR-AUC) were additionally computed in the validation dataset, and 95% confidence intervals for all metrics were obtained by bootstrap resampling (2000 replicates). In this study, we implemented feature extraction, model development and evaluation using the pandas [25], NumPy [26] and scikit-learn [27] libraries in Python 3.11 [28]. The reimplementation of the analysis pipeline was carried out in Python 3.13 with the assistance of an AI-based code generation tool, and the analysis code is provided as Supplementary Materials (see Supplementary Code). The number of latent variables of the final model was selected on the calibration dataset by 10-fold cross-validation over the grid 5, 10, 15, 17, 20 and 25; 17 latent variables gave a validation sensitivity, specificity and balanced accuracy of 0.84, 0.80 and 0.82, and 20 (the selected value) gave 0.83, 0.79 and 0.81, with an AUC of 0.88 in both cases; the choice therefore affects every metric by at most 0.01. The study design, the input data, the modeling parameters and the interpretation of the results were determined and verified by the authors. As a robustness check, the final model was additionally evaluated on the subset of the validation dataset whose cows do not occur in the calibration dataset, and the complete pipeline was rederived under a grouped split in which whole cows were assigned to either the calibration or the validation group; both analyses are reported in Supplementary Table S3. For the final model, two additional analyses were performed: the eight wavenumbers falling within the ν_3_ asymmetric stretching band of atmospheric CO_2_ were removed from the selected set, and the variable selection was repeated after excluding the whole 2300–2400 cm^−1^ interval from the candidate pool; SNV, MSC and Savitzky–Golay derivative transformation were also compared (Supplementary Table S4).

## 3. Results

### 3.1. Dataset Division and Characteristics of the Calibration Dataset for Ketosis Detection in Dairy Cows

Table 1 summarizes the dataset division of the dairy cow ketosis detection model and indicators such as daily milk yield, milk composition and somatic cell count. As shown in Table 1: (a) in the calibration dataset, we observed a significant difference in daily milk yield between healthy and ketotic cows; (b) no significant differences were observed for somatic cell count, somatic cell score, or differential somatic cell count; and (c) differences in milk composition between healthy and ketotic cows suggest corresponding differences in their MIR spectra. Therefore, it is possible to establish a detection model for healthy and ketotic cows based on milk MIR spectra.

### 3.2. Ketosis-Related Milk MIR Feature Wavenumbers

#### 3.2.1. Comparison of Selection Methods for Feature Wavenumbers

Table 2 summarizes the performance of the five feature wavenumber selection methods (NONE, MCUVE, CARS, Random Frog, and MFE) when combined with the five machine learning algorithms.

**Table 2:** Modeling methods and evaluation of MIR spectroscopy detection models for ketosis in dairy cows.

Modeling Conditions			Model Evaluation					
Feature Selection Method	Number of Wavenumbers	Modeling Algorithm	Cross-Validation			Validation		
			SENS	SPEC	Balanced Accuracy	SENS	SPEC	Balanced Accuracy
NONE	875	PLS-DA	0.75	0.74	0.74	0.76	0.74	0.75
		LASSO	0.71	0.76	0.74	0.72	0.77	0.74
		SVM	0.61	0.69	0.65	0.66	0.70	0.68
		RF	0.67	0.64	0.65	0.75	0.65	0.70
		ANN	0.65	0.59	0.62	0.68	0.64	0.66
MCUVE	6	PLS-DA	0.65	0.68	0.66	0.65	0.67	0.66
		LASSO	0.58	0.69	0.63	0.59	0.68	0.63
		SVM	0.50	0.50	0.50	0.50	0.50	0.50
		RF	0.54	0.59	0.56	0.57	0.60	0.59
		ANN	0.50	0.56	0.53	0.52	0.55	0.54
**CARS**	**104**	**PLS-DA**	**0.82**	**0.79**	**0.81**	**0.82**	**0.78**	**0.80**
		LASSO	0.69	0.81	0.75	0.71	0.79	0.75
		SVM	0.37	0.84	0.60	0.40	0.85	0.63
		RF	0.66	0.63	0.65	0.72	0.65	0.69
		ANN	0.60	0.66	0.63	0.65	0.69	0.67
Random Frog	92	PLS-DA	0.75	0.73	0.74	0.76	0.74	0.75
		LASSO	0.71	0.73	0.72	0.73	0.75	0.74
		SVM	0.35	0.83	0.59	0.38	0.85	0.61
		RF	0.68	0.62	0.65	0.75	0.65	0.70
		ANN	0.60	0.70	0.65	0.65	0.74	0.69
MFE	116	PLS-DA	0.79	0.77	0.78	0.82	0.76	0.79
		LASSO	0.72	0.76	0.74	0.78	0.78	0.78
		SVM	0.36	0.83	0.59	0.39	0.84	0.62
		RF	0.68	0.66	0.67	0.74	0.65	0.70
		ANN	0.55	0.68	0.62	0.65	0.73	0.69

Note: SENS, SPEC, AUC and balanced accuracy denote the sensitivity, specificity, the area under the receiver operating characteristic curve and the mean of sensitivity and specificity, respectively. Bold type is used to highlight the optimal model combination. The table reports the original screening run, in which the 25 combinations were compared on sensitivity, specificity and balanced accuracy; the AUC was not computed for the combinations other than CARS–PLS-DA, and could not be recomputed for the remaining ones because their selection outputs were not archived. The CARS–PLS-DA combination is the final model, and its complete metric set, including a validation AUC of 0.88 and a PR-AUC of 0.51, is given in Table 3.

**Table 3:** Additional performance metrics of the PLS-DA model based on 104 CARS-selected wavenumbers in the validation dataset (1182 healthy and 174 ketotic samples; ketosis prevalence 12.8%).

Metric	Estimate	95% CI
Sensitivity	0.83	0.78–0.89
Specificity	0.79	0.77–0.82
Balanced accuracy	0.81	0.78–0.84
AUC	0.88	—
PR-AUC	0.51	—
Precision (PPV)	0.37	0.32–0.42
Negative predictive value	0.97	0.96–0.98
F1-score	0.51	0.46–0.56
MCC	0.46	0.41–0.51

Note: Metrics were computed from a reconstruction of the original PLS-DA analysis, using the original modeling dataset and the 104 wavenumbers selected by CARS. The original modeling script is no longer available; the reconstruction reproduced the sensitivity, specificity and balanced accuracy reported in Table 2 to within 0.01 (0.83, 0.79 and 0.81 vs. 0.82, 0.78 and 0.80), and the analysis code is provided as Supplementary Materials. The number of latent variables was selected on the calibration dataset by 10-fold cross-validation; 17 latent variables gave a validation sensitivity, specificity and balanced accuracy of 0.84, 0.80 and 0.82, and 20 (the value selected) gave 0.83, 0.79 and 0.81, with an AUC of 0.88 in both cases.

As shown in Table 2: (a) compared with NONE, the MCUVE, CARS, Random Frog and MFE selection methods reduced the number of MIR wavenumbers, with the number of feature wavenumbers screened by these selection methods being 6, 104, 92 and 116, respectively; (b) The MCUVE selection method screened out 6 spectral feature wavenumbers, but it yielded the lowest sensitivity, specificity and balanced accuracy in the validation dataset; (c) compared with NONE (875 wavenumbers), the CARS, Random Frog and MFE selection methods improved the prediction performance of the MIR model, where the highest values of sensitivity, specificity and balanced accuracy of these selection methods in the validation dataset were 0.82, 0.85 and 0.80, respectively; and (d) CARS selected substantially fewer wavenumbers than the raw spectrum (104 vs. 875) and fewer than the MFE method (116), and, with a compactness comparable to Random Frog (92), achieved the best predictive performance in the validation dataset as measured by the balanced accuracy. In this study, we established a ketosis detection model using the 104 feature wavenumbers screened by CARS.

#### 3.2.2. Strategy of CARS for Selecting Feature Wavenumbers

**Figure 2:**
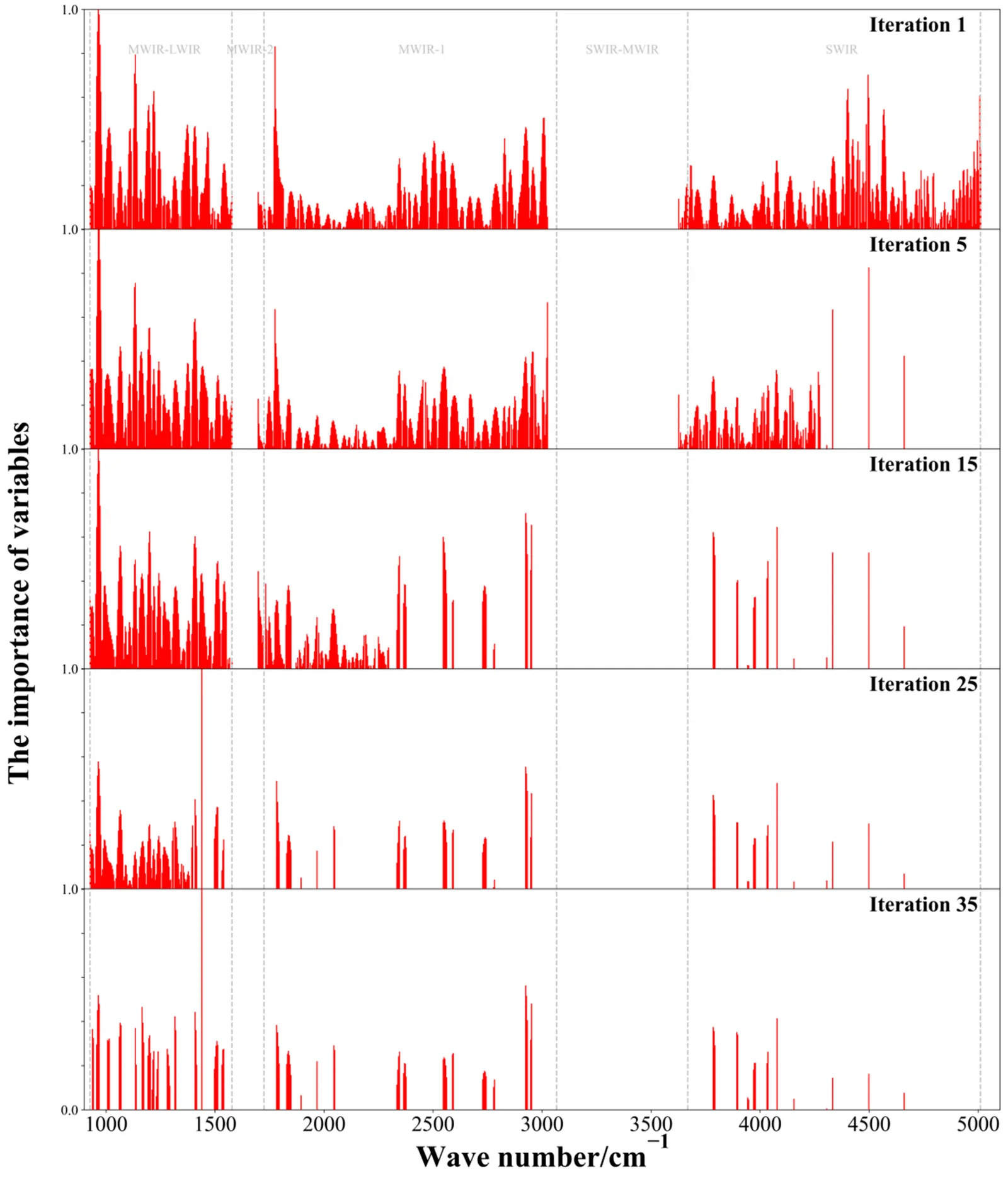
Changes in the number and importance of feature wavenumbers during CARS iterations; iterations 1, 5, 15, 25 and 35 are shown. The subset with the lowest cross-validated RMSECV, which was used for the final model, contained 104 wavenumbers (Supplementary Table S1).

Figure 2 shows the changes in the number and importance of MIR feature wavenumbers across the elimination iterations within the CARS run whose selection was used for the final model. As the iteration progresses, wavenumbers with redundancy or low contribution are gradually removed, resulting in a significant reduction in the number of ultimately retained feature wavenumbers. The importance indices of most non-feature wavenumbers are close to 0, indicating that CARS can effectively compress the MIR variable space and retain information related to ketosis discrimination.

### 3.3. Algorithms for the Ketosis Detection Model

Table 2 summarizes the effects of using five model development algorithms (PLS-DA, LASSO, SVM, RF and ANN) to establish ketosis detection models in dairy cows. As shown in Table 2, PLS-DA combined with different feature wavenumber selection methods achieved the highest balanced accuracy and the highest sensitivity in both the cross-validation and validation datasets; the highest validation specificity (0.85) was obtained by combinations using SVM. In this study, we established a model using no feature selection (NONE), the CARS method for feature wavenumber selection, the PLS-DA algorithm and 104 feature wavenumbers. Our MIR-based ketosis detection model, denoted as the “PLS-DA model based on 104 CARS-selected wavenumbers”, achieved satisfactory performance. The sensitivity, specificity and balanced accuracy of this model in the validation dataset are 0.83, 0.79 and 0.81, and the additional performance metrics, including an AUC of 0.88 and a PR-AUC of 0.51, are reported in Table 3, and the corresponding ROC and precision–recall curves are provided in Supplementary Figure S1. The robustness of the final model to the cow-level structure of the data was examined in two ways (Supplementary Table S3). Applied unchanged to the 1060 validation samples whose cows do not occur in the calibration dataset, the model gave a sensitivity of 0.84, a specificity of 0.78, a balanced accuracy of 0.81 and an AUC of 0.88 (TP = 111, FP = 202, TN = 726, FN = 21); the same model on the complete validation set gave 0.83, 0.79, 0.81 and 0.88, and on the 296 samples from cows shared with the calibration set the AUC was 0.88. The entire pipeline was then rederived under a grouped split in which no cow occurred in both groups, the calibration group containing 852 samples from 647 cows and the validation group 1354 samples from 924 cows; CARS was rerun on the calibration group only and retained 54 wavenumbers, and the resulting model achieved an AUC of 0.80 and a balanced accuracy of 0.72 on the held-out cows. With matched numbers of latent variables, the grouped split lowered the AUC by 0.005–0.028 relative to the sample-level control, whereas the difference with respect to the archived model is attributable to the variable-selection step: the archived run retained 104 wavenumbers and the reimplemented CARS 45–54, while the ten archived runs of CARS selected between 38 and 176.

The sensitivity of the final model to the atmospheric CO_2_ band and to spectral preprocessing was also examined (Supplementary Table S4). Of the 104 wavenumbers selected by CARS, eight (points 605–616, 2334.09–2376.53 cm^−1^) lie within the ν_3_ asymmetric stretching band of atmospheric CO_2_ and 14 (points 981–1077, 3784.70–4155.07 cm^−1^) lie at the high-frequency edge of the water O–H band. Removing the eight CO_2_-band wavenumbers and leaving everything else unchanged gave a sensitivity of 0.81, a specificity of 0.79, a balanced accuracy of 0.80 and an AUC of 0.86, against 0.83, 0.79, 0.81 and 0.88 for the complete set, so the band contributes about 0.02 in AUC and is not required for the model to perform. Excluding 2300–2400 cm^−1^ from the candidate pool before CARS (848 candidates) gave an AUC of 0.83 and a balanced accuracy of 0.74 under the original split, and an AUC of 0.79 and a balanced accuracy of 0.74 under the cow-level grouped split. With the 104-wavenumber selection and the split held fixed, SNV, MSC, a Savitzky–Golay first or second derivative (window 11, polynomial order 2) and SNV followed by the first derivative gave AUC values between 0.88 and 0.89 and balanced accuracies between 0.81 and 0.82; the same comparison on the CO_2_-free 96-wavenumber set gave AUC values between 0.86 and 0.87. No preprocessing method materially improved the discrimination.

### 3.4. Feature Wavenumbers Related to Ketosis in Dairy Cows and Their Corresponding Functional Groups and Substance Components

**Figure 3:**
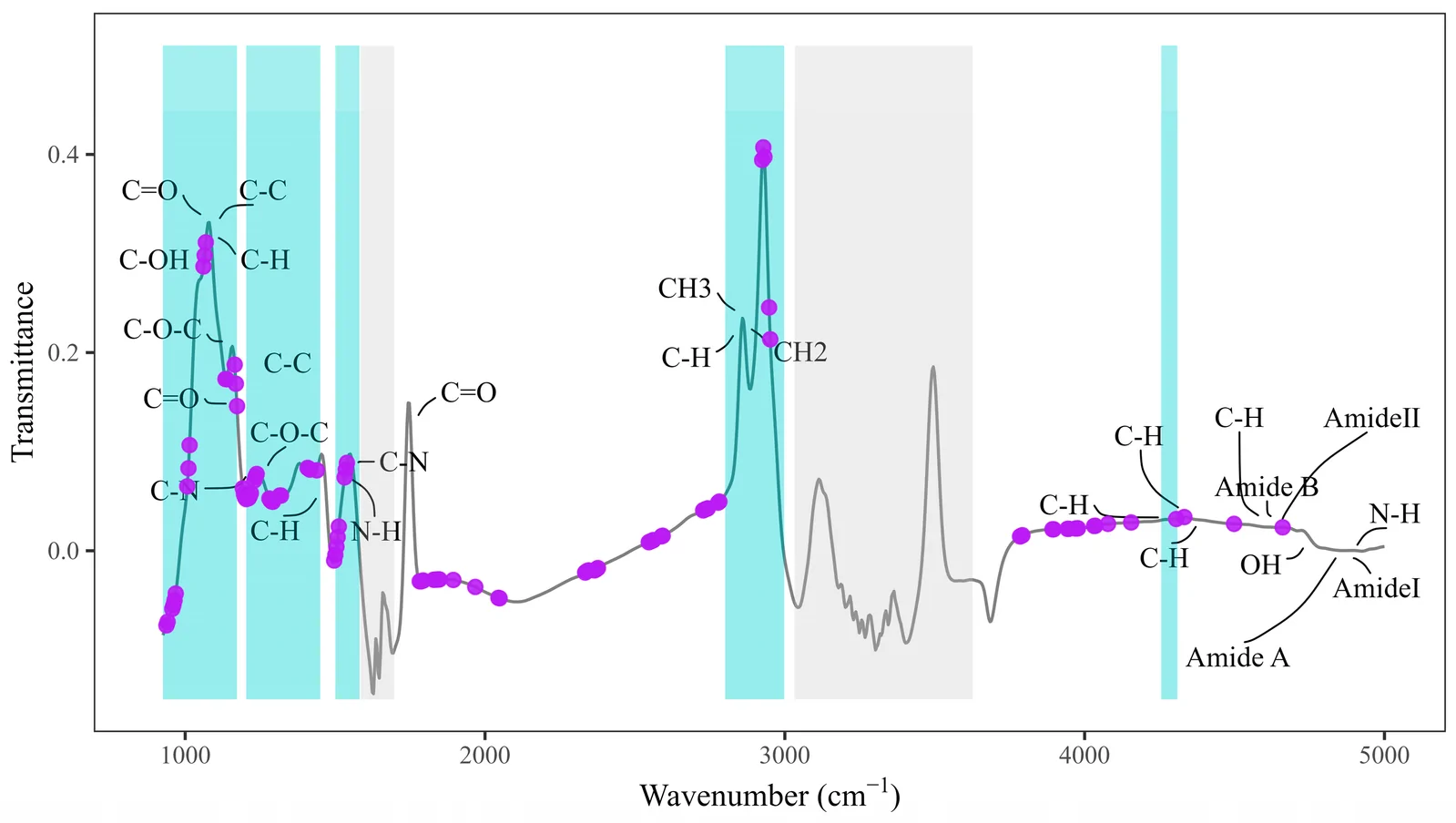
Distribution of ketosis-related feature wavenumbers selected by CARS in the MIR spectrum. The purple dots indicate the 104 feature wavenumbers selected by CARS. The blue area represents the spectral region associated with milk components such as fat, protein and lactose. The gray area represents the water absorption regions in the spectrum.

Figure 3 shows the distribution of the 104 ketosis-related feature wavenumbers selected by CARS in the MIR spectrum. Among them, 17 feature wavenumbers are located in the range of 925.92–1172.83 cm^−1^, which is an important absorption region for milk protein, milk fat, and lactose; 20 feature wavenumbers are located in the range of 1192.12–1450.61 cm^−1^, and 8 feature wavenumbers are located in the range of 1496.90–1581.78 cm^−1^. These two regions are mainly related to milk protein absorption; 5 feature wavenumbers are located in the range of 2800.91–2997.67 cm^−1^, which is one of the milk fat absorption regions; the remaining 54 feature wavenumbers are distributed in the ranges of 1701.38–2797.05 cm^−1^ (36 wavenumbers) and 3734.54–4660.46 cm^−1^ (18 wavenumbers). This indicates that the spectral information related to ketosis [29] comes from both the regions of conventional milk components and the spectral intervals that have not been fully assigned. The upper limits of these intervals are the theoretical absorption-region boundaries reported in the literature rather than the extrema of the selected wavenumbers, the highest of which are 1172.83, 1439.03, 1539.34 and 2951.37 cm^−1^, respectively.

## 4. Discussion

In studies on the prediction of milk ketone bodies and ketosis detection models using parameters such as milk fat percentage, milk protein percentage and milk yield, the correlation between actual and predicted values of acetone and β-hydroxybutyrate in milk ranged from 0.64 to 0.80 [11,12]. The sensitivity of ketosis detection models ranged from 0.69 to 0.75 and specificity ranged from 0.73 to 0.95 [11,13]. These studies suggest that it is feasible to use milk components as parameters for ketosis detection models. MIR spectroscopy of milk is widely used for batch detection of milk fat, protein and lactose percentages. The mid-infrared spectrum of milk measured by the FOSS milk component analyzer includes 1060 MIR wavenumbers in the range of 925–5011 cm^−1^. The currently known spectral regions for milk fat, protein and lactose include wavenumber ranges such as 925.92–1172.83, 1192.12–1450.61, 1500.76–1581.78 and 2800.91–2997.67 cm^−1^ [14,15,16]; these limits are the theoretical absorption-region boundaries reported in the literature and not the extrema of the selected wavenumbers. Compared with single milk components or ketone body indicators, the MIR spectrum covers 1060 wavenumbers in the 925–5011 cm^−1^ range. In addition to the known absorption regions for milk fat, milk protein and lactose, it also contains many spectral segments that have not been fully assigned, which may provide additional information for ketosis discrimination.

In this study, we observed increased milk fat percentage and fat-to-protein ratio and a decreased milk protein percentage and lactose percentage in ketotic cows, consistent with the physiological characteristics of a negative energy balance and enhanced fat mobilization during ketosis [30]. The 104 feature wavenumbers selected by CARS are distributed not only in the regions related to milk fat, milk protein and lactose but also in unknown or insufficiently assigned regions such as 1701.38–2797.05 cm^−1^ and 3734.54–4660.46 cm^−1^. Therefore, we speculate that the MIR model for ketosis discrimination may utilize both the changes in conventional milk components and more complex metabolism-related spectral information.

Through feature wavenumber selection and modeling algorithms, our established ketosis detection model, denoted as the “PLS-DA model based on 104 CARS-selected wavenumbers”, performed well. The sensitivity, specificity and balanced accuracy of this model in the validation dataset were 0.83, 0.79 and 0.81, and the additional metrics, including an AUC of 0.88 and a PR-AUC of 0.51, are reported in Table 3. Our model achieved higher sensitivity while maintaining comparable overall discrimination. For example, Satoła and Bauer established a ketosis detection model using parameters such as parity, days in milk, fat-to-protein ratio, acetone, β-hydroxybutyric acid and lactose percentage, with sensitivity ranging from 0.74 to 0.75 and specificity from 0.73 to 0.78 [13]. The specificity of our model (0.79) was lower than that reported by De Roos et al. (0.95), whereas its sensitivity (0.83) was higher than the 0.69–0.70 reported in that study [11]. Compared with other studies, we used a limited number of MIR features as model parameters, which facilitates application and enhances generalization ability. This variable-selection strategy is consistent with evidence that targeted MIR regions can retain disease-related information [29]. From an application perspective, the high sensitivity of our model is beneficial for identifying potential ketosis cases in DHI testing, whereas the specificity still needs further improvement to reduce false alarms and the associated costs of confirmatory testing. Because the specificity of the model is 0.79, the proportion of false positives remains substantial at the prevalence typical of commercial herds, and the model is therefore best used as a pre-selection step whose positive results are confirmed by blood BHB measurement rather than as a stand-alone diagnostic test. Recent work has also demonstrated the utility of milk MIR spectra for predicting blood BHB at scale and generating phenotypes for genetic evaluation [31].

We acknowledge several limitations in this study. First, the ketosis labels were derived from farm health diagnosis records. The records do not state the BHB threshold or the meter type used on each farm, so inter-farm and inter-operator variability in the diagnosis, together with the subclinical cases that may have been missed because of testing frequency or incomplete records, constitutes an additional source of labeling noise. Second, our samples came from 12 farms in central China, and the generalization ability of the model across different regions, feeding management systems, and instrument platforms [32] still needs to be verified using an external independent population. Third, because the number of ketotic samples was limited, the samples were randomly divided into the calibration and validation datasets at the sample level rather than at the animal level, and the informative wavenumbers were selected on the calibration dataset as a whole rather than within each cross-validation fold. We quantified both issues (Supplementary Table S3). Evaluating the final model on the 1060 validation samples whose cows do not occur in the calibration dataset left the AUC and the balanced accuracy essentially unchanged (0.88 and 0.81 on both), so the cow-level overlap of the original split introduced no detectable optimistic bias. Rederiving the whole pipeline under a grouped split in which no cow occurred in both groups gave an AUC of 0.80 and a balanced accuracy of 0.72, i.e., at most 0.03 below the sample-level control, the residual difference being attributable to the variable-selection step rather than to the split. The variable-selection step, however, was still applied to the calibration dataset as a whole rather than within each cross-validation fold, so the internally cross-validated estimates may remain slightly optimistic; the external validation metrics reported here are not affected, because the wavenumbers were selected exclusively on the calibration dataset and applied unchanged to the validation dataset. Fourth, no spectral preprocessing (such as scatter correction or derivative transformation) was applied, because the aim was to evaluate the model under the conditions in which DHI spectra are routinely produced. We have since tested SNV, MSC and Savitzky–Golay derivative transformation on the final model and found that they change the discrimination by less than 0.01 in AUC (Supplementary Table S4); whether they would be beneficial for spectra produced under different conditions remains to be tested. Fifth, eight of the 104 selected wavenumbers (points 605–616, 2334.09–2376.53 cm^−1^) fall within the ν_3_ asymmetric stretching band of atmospheric CO_2_, and 14 (points 981–1077, 3784.70–4155.07 cm^−1^) lie at the high-frequency edge of the water O–H band. Removing the former changes the AUC by about 0.02 and makes the model slightly worse, and excluding the entire 2300–2400 cm^−1^ interval before variable selection does not improve the discrimination (AUC 0.83); we have therefore reported the model as it was derived, but readers should be aware that the candidate pool was not restricted in this respect. In future studies, we could optimize the model threshold, generalization performance and biological interpretability by incorporating continuous blood BHB measurements, longitudinal sampling and external multi-center data [8].

## 5. Conclusions

Based on DHI milk samples and ketosis diagnosis records from 12 large-scale dairy farms in central China, we compared the combined effects of five MIR feature wavenumber selection methods and five model development algorithms. Our results showed that the ketosis prediction model established by combining the 104 feature wavenumbers screened by CARS with PLS-DA performed best, with a sensitivity, specificity and balanced accuracy of 0.83, 0.79 and 0.81 in the validation set, and an AUC of 0.88. This model has the potential for high-throughput ketosis screening and batch acquisition of phenotypes in the DHI system, but we still need to further verify and optimize it using data from larger scales, more regions and independent populations.

## Figures and Tables

**Table 1:** Milk components and somatic cell indicators in the MIR model development and validation datasets for ketosis in dairy cows.

Health Status	Calibration Dataset		Validation Dataset	
	Healthy	Ketosis	Healthy	Ketosis
Number of records	425	425	1182	174
Daily milk yield, kg	38.40 (1.30) ^a^	35.30 (1.32) ^b^	36.10 (0.84) ^NS^	35.10 (1.25) ^NS^
Milk protein, %	3.21 (0.04) ^A^	3.09 (0.04) ^B^	3.24 (0.03) ^A^	3.06 (0.04) ^B^
Milk fat, %	4.25 (0.13) ^A^	4.73 (0.14) ^B^	4.43 (0.08) ^A^	5.06 (0.12) ^B^
Milk lactose, %	5.08 (0.03) ^A^	4.99 (0.03) ^B^	5.07 (0.02) ^A^	4.98 (0.03) ^B^
Total solids, %	13.10 (0.14) ^NS^	13.30 (0.14) ^NS^	13.30 (0.09) ^a^	13.60 (0.13) ^b^
Fat-to-protein ratio	1.33 (0.05) ^A^	1.53 (0.05) ^B^	1.38 (0.03) ^A^	1.66 (0.04) ^B^
Somatic cell count, 10^3^ cells/mL	207.00 (71.70) ^NS^	242.00 (72.90) ^NS^	201.00 (36.10) ^NS^	266.00 (54.00) ^NS^
Somatic cell score	2.72 (0.20) ^NS^	2.84 (0.20) ^NS^	3.02 (0.12) ^NS^	3.28 (0.18) ^NS^
Differential somatic cell count, %	39.30 (3.81) ^NS^	42.20 (3.86) ^NS^	45.90 (2.42) ^NS^	46.30 (3.62) ^NS^

Note: Data are presented as least squares means ± standard errors. Multiple comparisons were performed using the Tukey adjustment in the lsmeans package. Superscript letters indicate significant differences between healthy cows and cows with ketosis. Specifically, no superscript letter (NS: No Significant) denotes no significant difference (*p* > 0.05); different lowercase letters denote a significant difference (*p* < 0.05); and different uppercase letters denote a highly significant difference (*p* < 0.01).

## Data Availability

Restrictions apply to the availability of these data. Data were obtained from the Henan Dairy Herd Improvement Center and the 12 participating commercial dairy farms and are available from the authors with the permission of the third party.

## Supplementary Materials

The following supporting information can be downloaded at: https://www.mdpi.com/article/10.3390/ani16203196/s1, Table S1: The 104 feature wavenumbers selected by CARS; Table S2: Hyperparameter settings used in the analysis; Table S3: Cow-level analyses of the final model (metrics on the strictly non-overlapping subset, and re-derivation under a grouped split by unique cow); Table S4: Sensitivity of the final model to the atmospheric CO_2_ band and to spectral preprocessing; Figure S1: ROC and precision–recall curves of the final model; Data S1: CARS selection matrix (875 candidate wavenumbers × 10 runs); Supplementary Code: reproduction of the analysis pipeline (scripts, requirements and example output).

## Abbreviations

The following abbreviations are used in this manuscript:

ANN	Artificial neural network
AUC	Area under the receiver operating characteristic curve
CARS	Competitive Adaptive Reweighted Sampling
FT-MIR	Fourier transform mid-infrared spectrometry
ICAR	International Committee for Animal Recording
LASSO	Least absolute shrinkage and selection operator
MCC	Matthews correlation coefficient
MCUVE	Monte Carlo Uninformative Variable Elimination
MFE	Multiple Feature-Spaces Ensemble
MIR	Mid-infrared spectra
NPV	Negative predictive value
PLS-DA	Partial least squares discriminant analysis
PPV	Positive predictive value
PR-AUC	Precision–recall area under the curve
RF	Random forest
SENS	Sensitivity
SPEC	Specificity
SVM	Support vector machine
