# Supplementary material for: Construction and Evaluation of a Ketosis Prediction Model Based on Milk MIR Spectra and Feature Wavenumber Selection in Dairy Cows
Source: Animals (Basel). 2026 Oct 12;16(20):3196. doi: 10.3390/ani16203196 (PMC13700896; doi:10.3390/ani16203196)
Supplement: Supplementary file 1 [file animals-16-03196-s001.zip › Supplementary_Figure_S1-ROC and precision–recall curves of the final model.pdf]

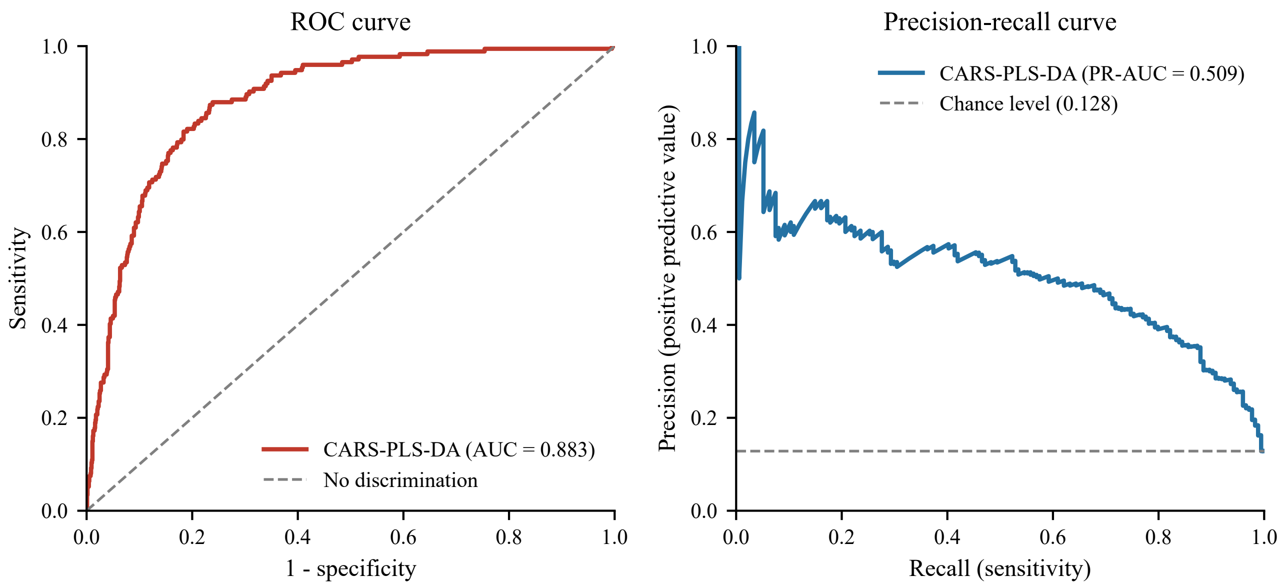
ROC curve
1.0
0.8
0.6
0.4
0.2
0.0
Sensitivity
0.0
0.2
0.4
0.6
0.8
1.0
1 - specificity
CARS-PLS-DA (AUC = 0.883)
No discrimination
Precision-recall curve
Precision (positive predictive value)
Recall (sensitivity)
CARS-PLS-DA (PR-AUC = 0.509)
Chance level (0.128)
